# Supplementary figures and images for: Rapid and precise genome engineering in a naturally short-lived vertebrate
Source: eLife. 2023 May 16;12:e80639. doi: 10.7554/eLife.80639 (PMC10188113; doi:10.7554/eLife.80639)

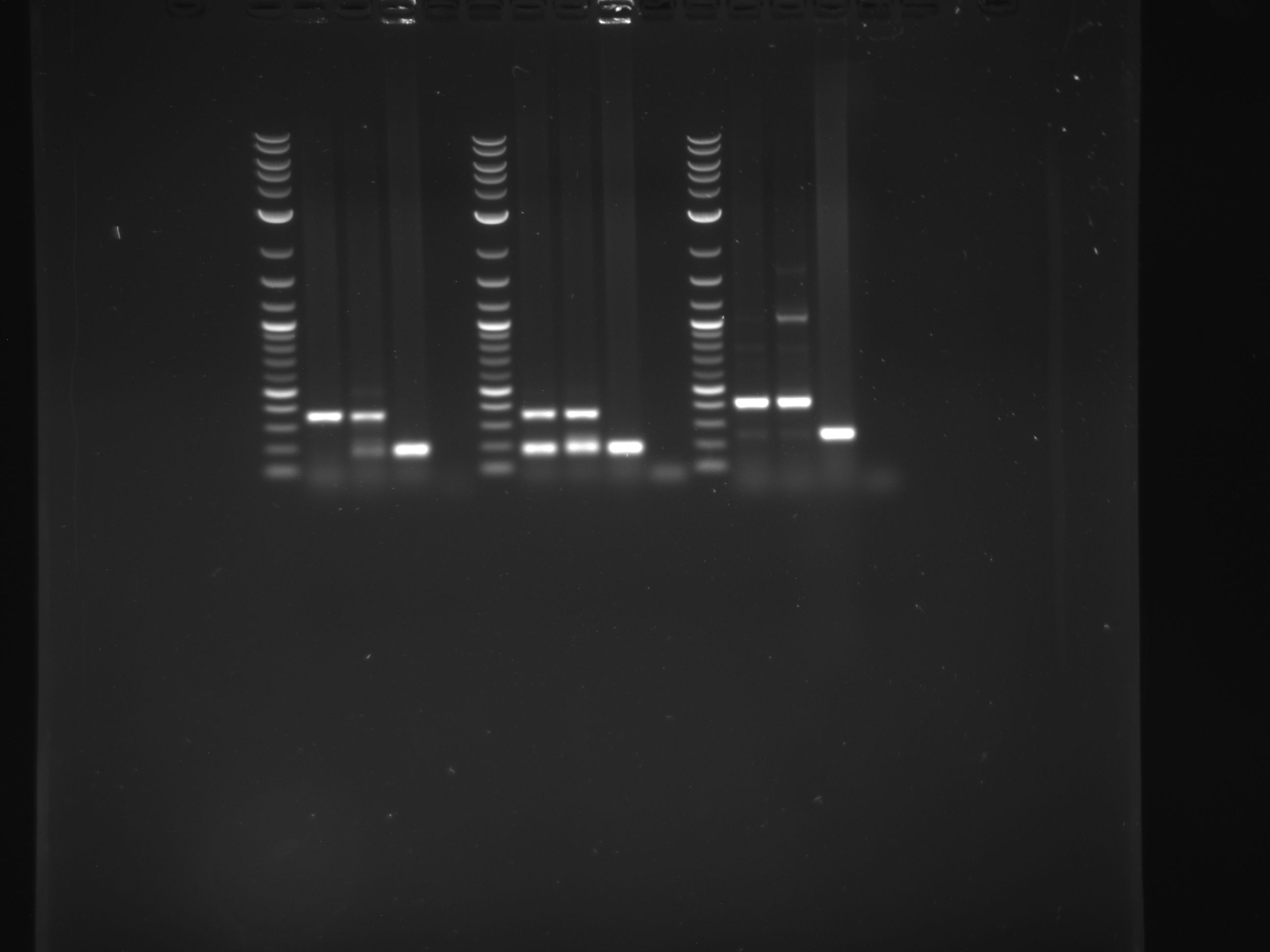

Supplement: Figure 1—source data 1. [file elife-80639-fig1-data1.zip › Figure1-Source Data1.jpg]

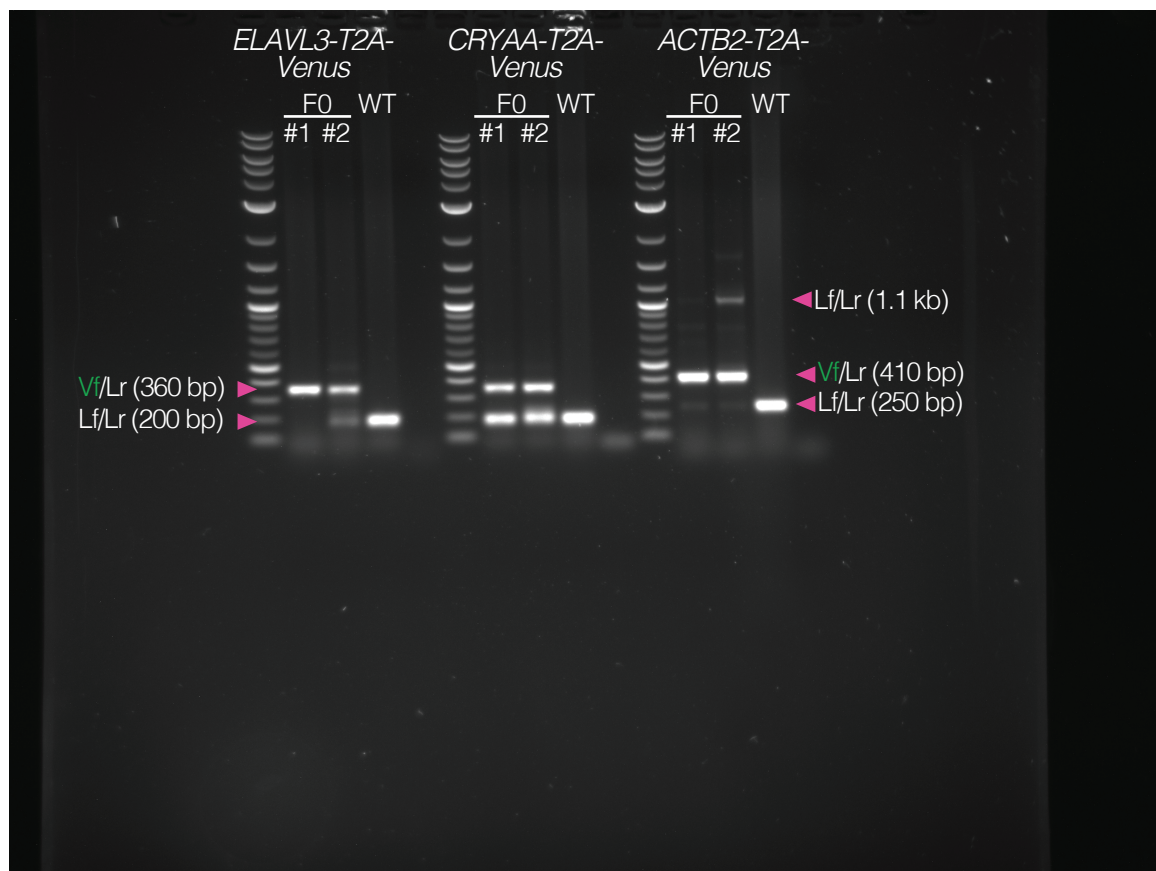

Supplement: Figure 1—source data 2. [file elife-80639-fig1-data2.zip › Figure1-Source Data2.pdf]

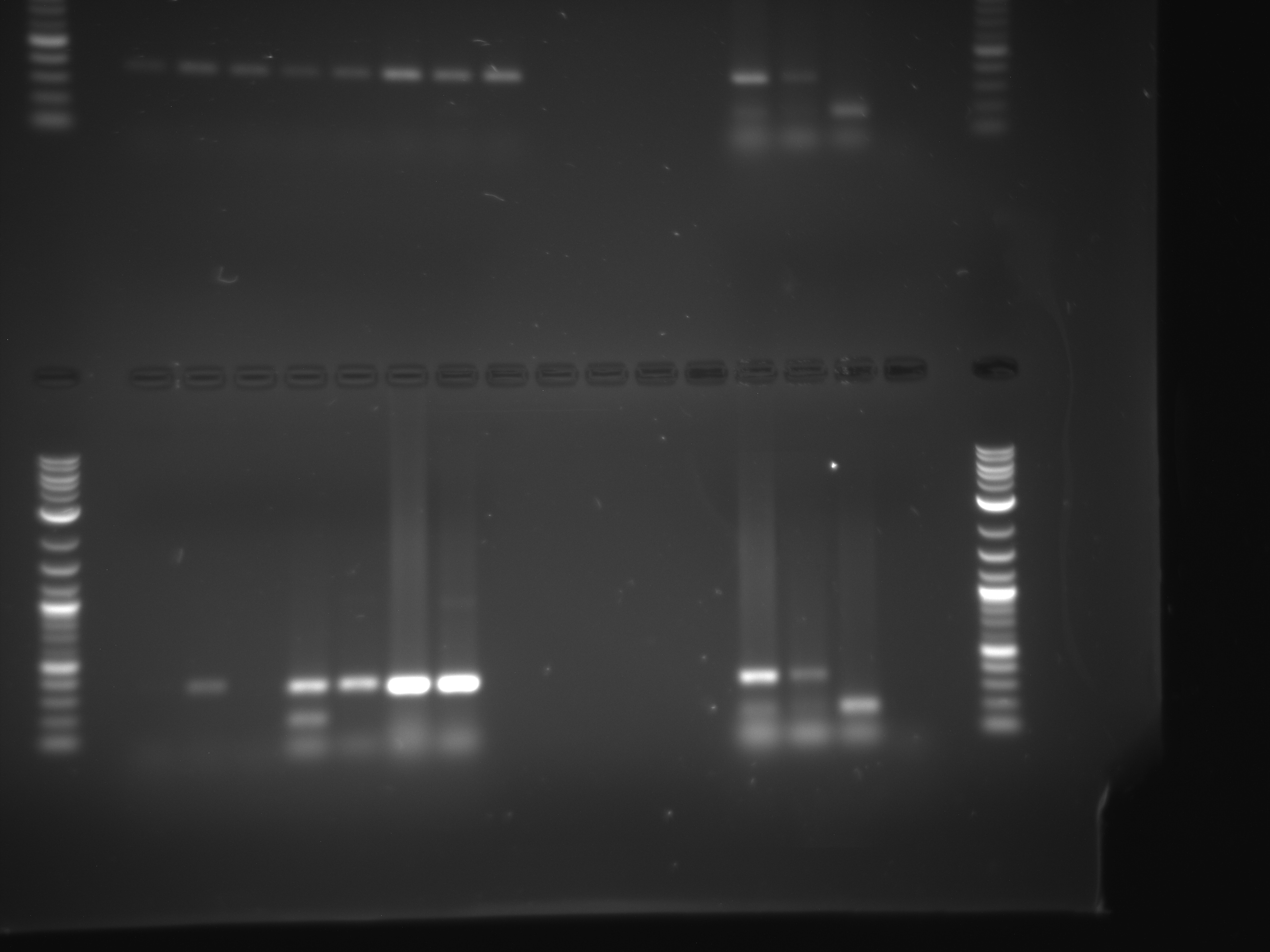

Supplement: Figure 2—source data 1. [file elife-80639-fig2-data1.zip › Figure2-Source Data1.jpg]

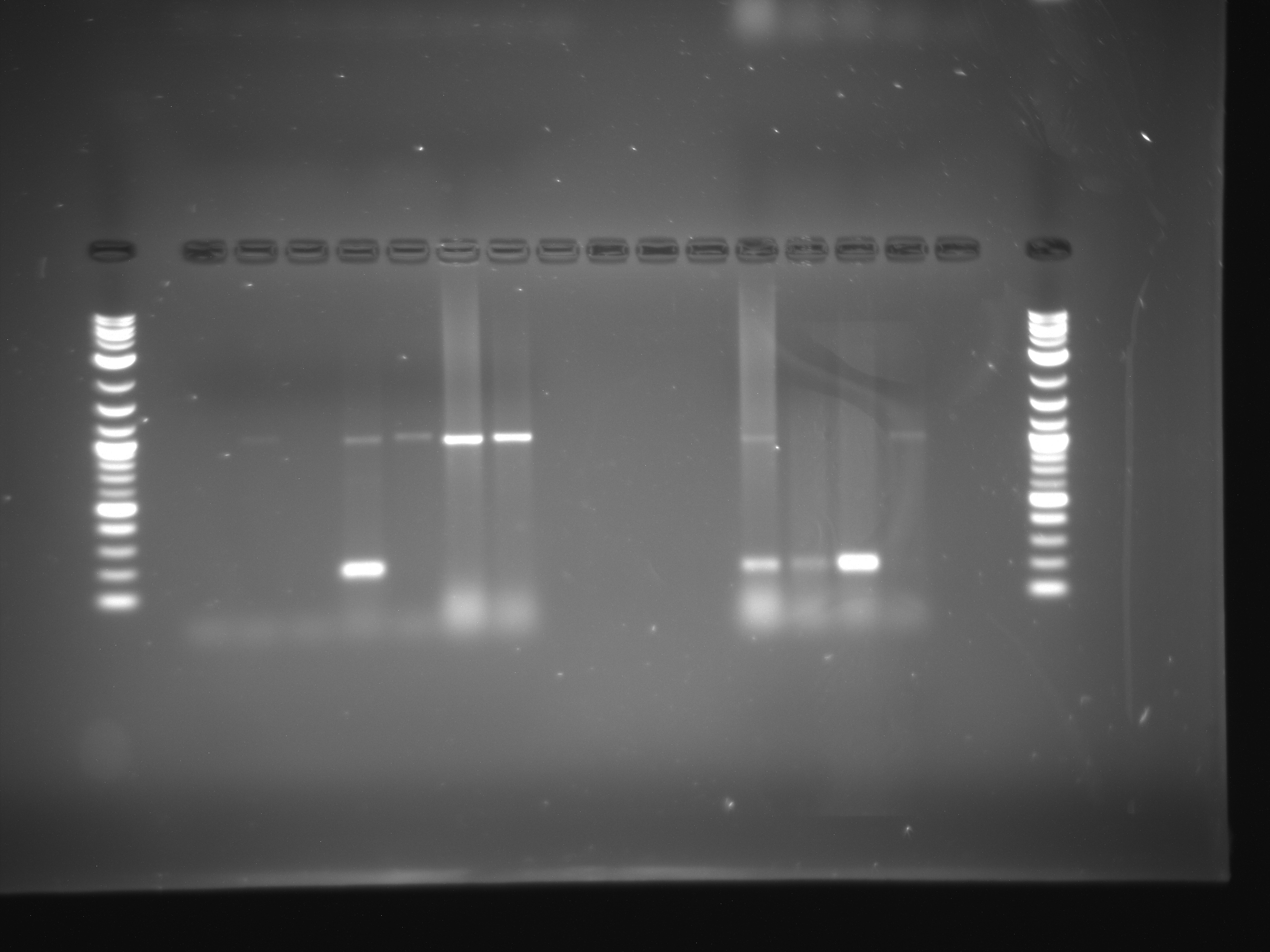

Supplement: Figure 2—source data 2. [file elife-80639-fig2-data2.zip › Figure2-Source Data2.jpg]

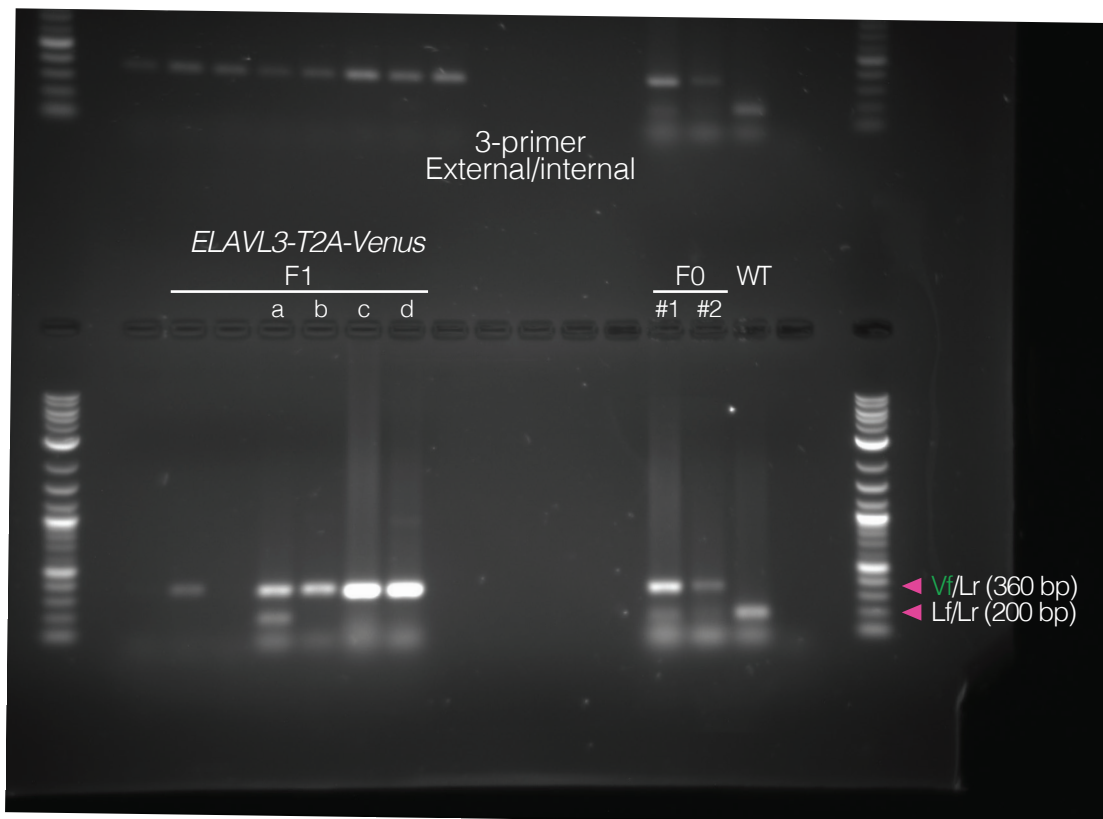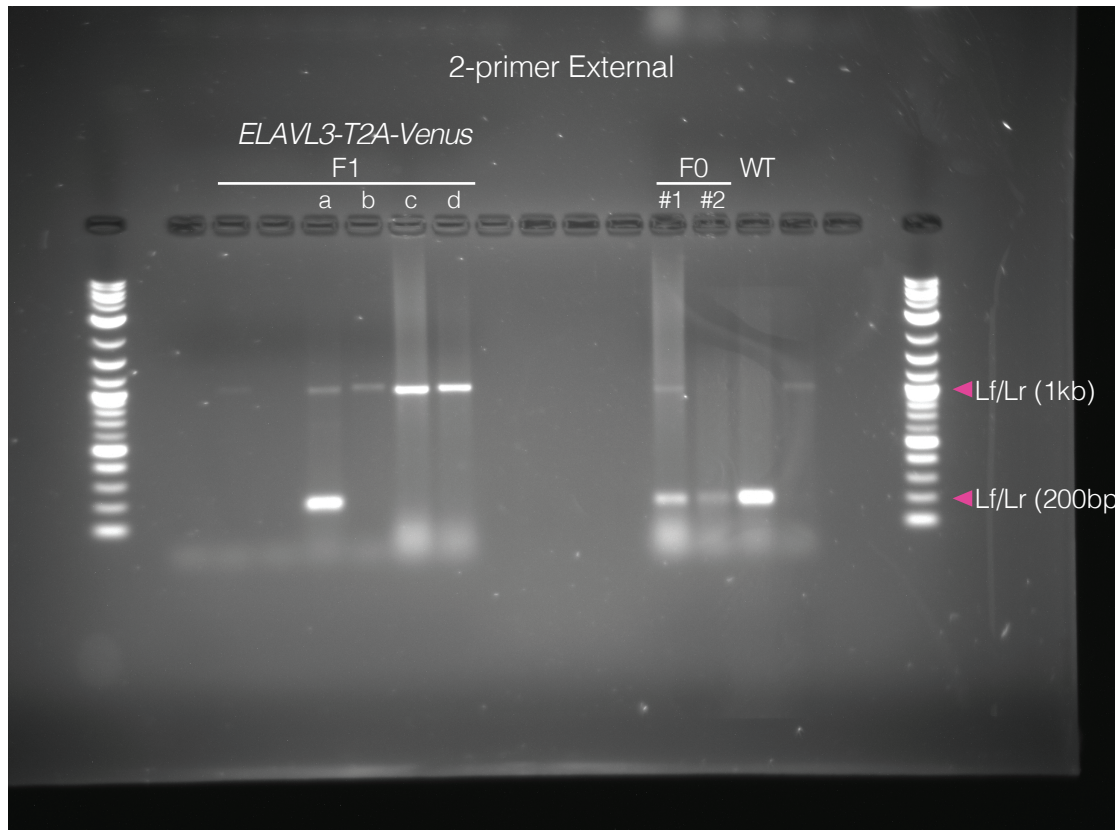

Supplement: Figure 2—source data 3. [file elife-80639-fig2-data3.zip › Figure2-Source Data3.pdf]

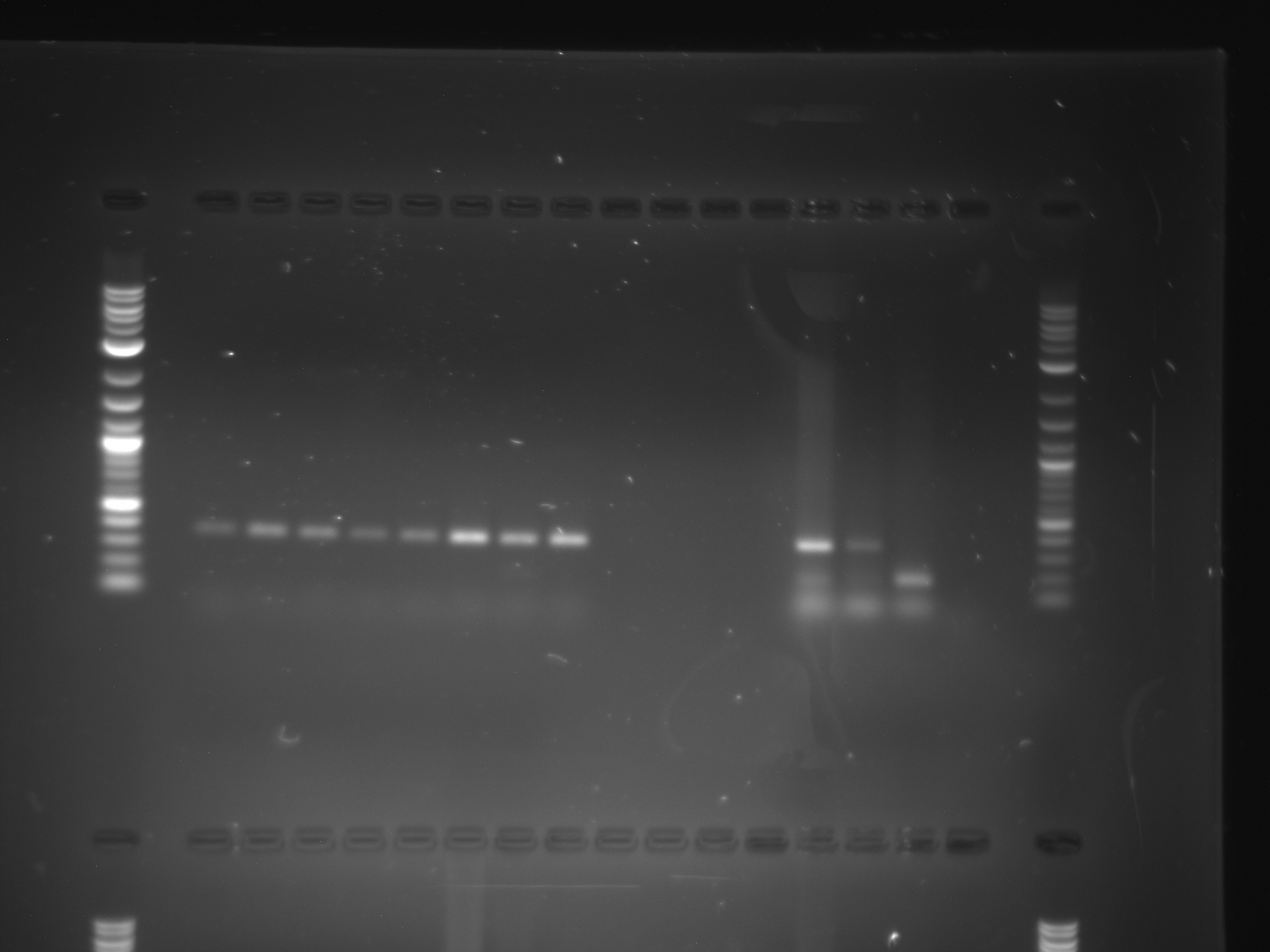

Supplement: Figure 2—figure supplement 1—source data 1. [file elife-80639-fig2-figsupp1-data1.zip › Figure2-figure supplement 1-Source Data1.jpg]

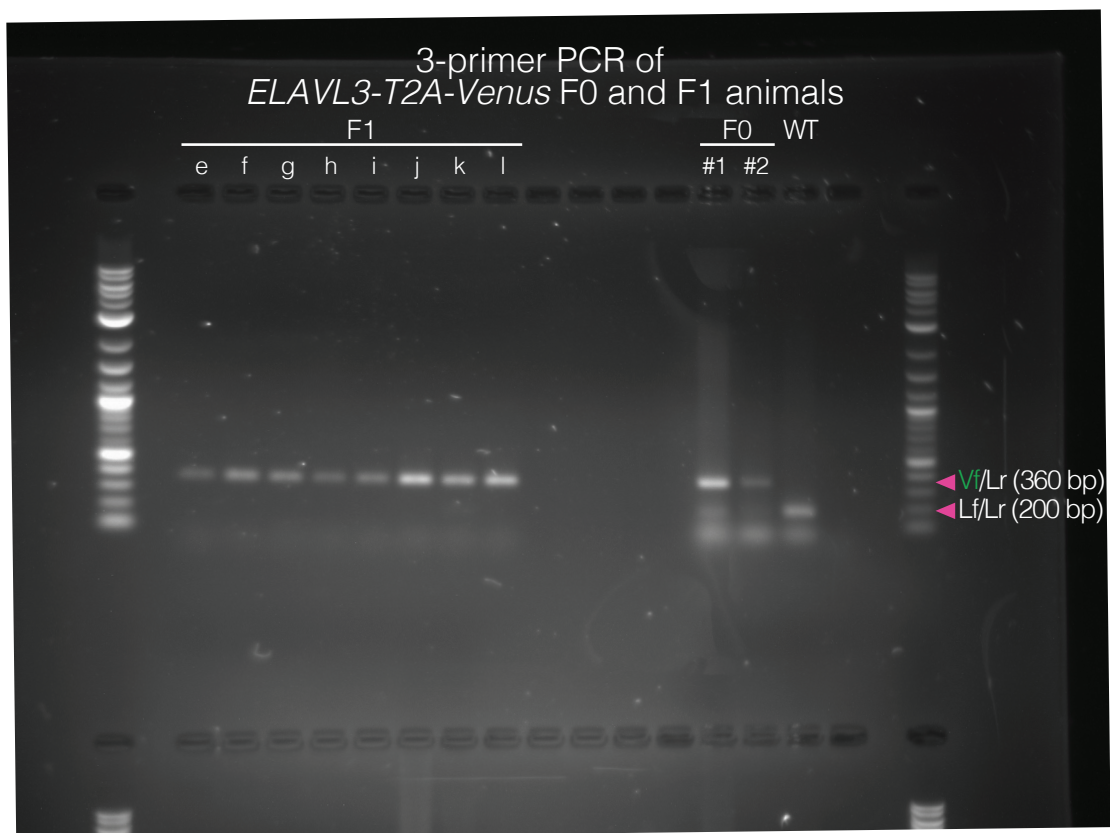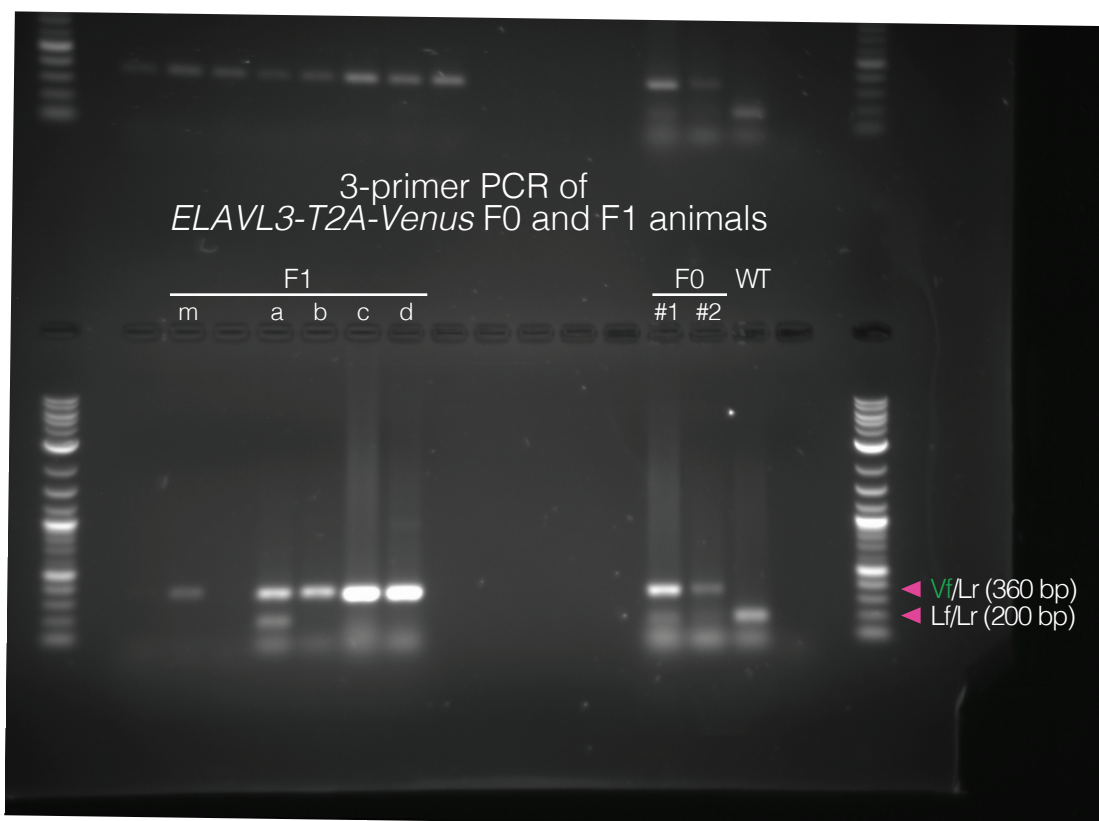

Supplement: Figure 2—figure supplement 1—source data 2. [file elife-80639-fig2-figsupp1-data2.zip › Figure2-figure supplement 1-Source Data2.pdf]

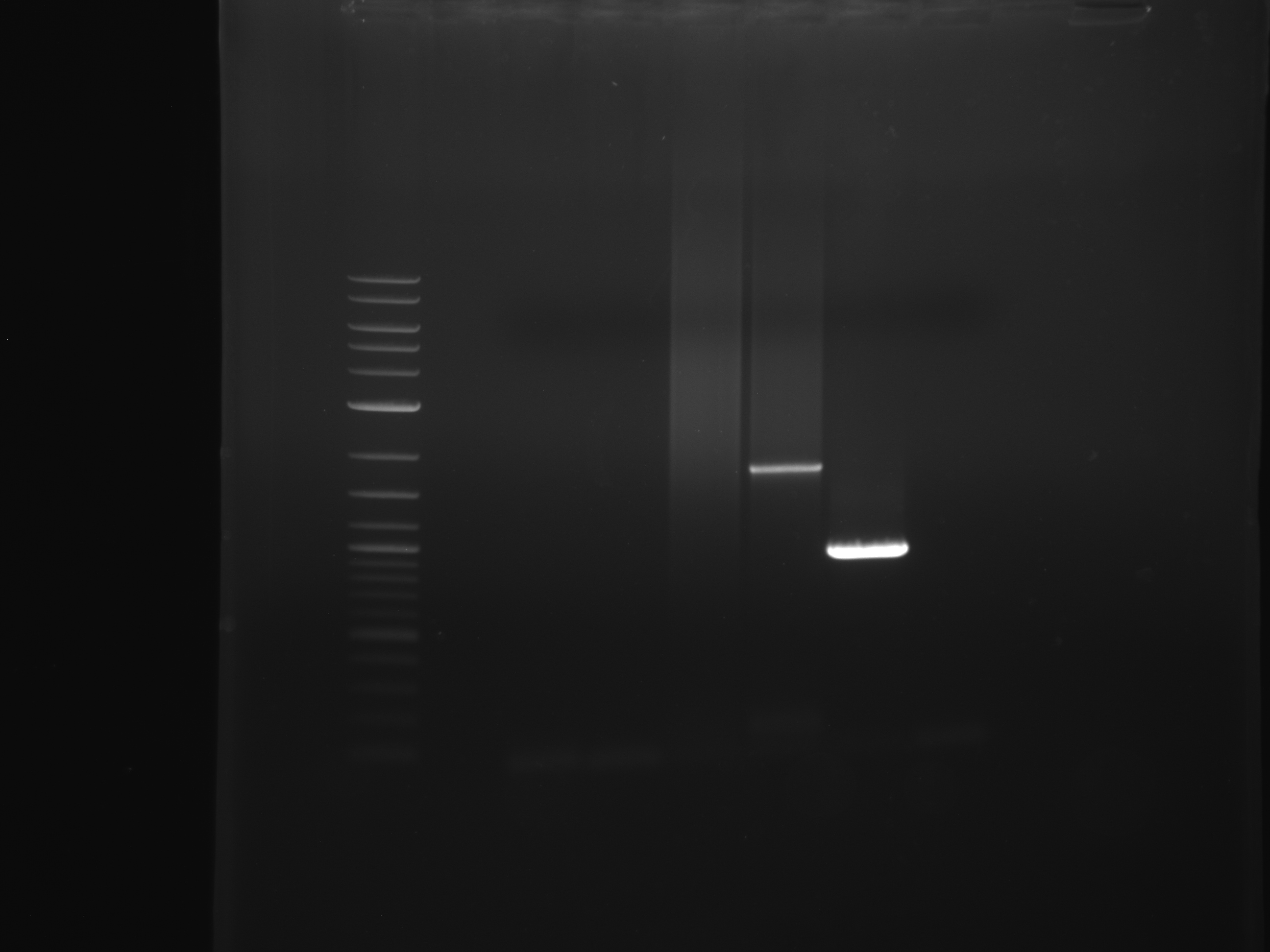

Supplement: Figure 2—figure supplement 1—source data 3. [file elife-80639-fig2-figsupp1-data3.zip › Figure2-figure supplement 1-Source Data3.jpg]

External PCR of  
*ELAVL3-T2A-Venus* F1

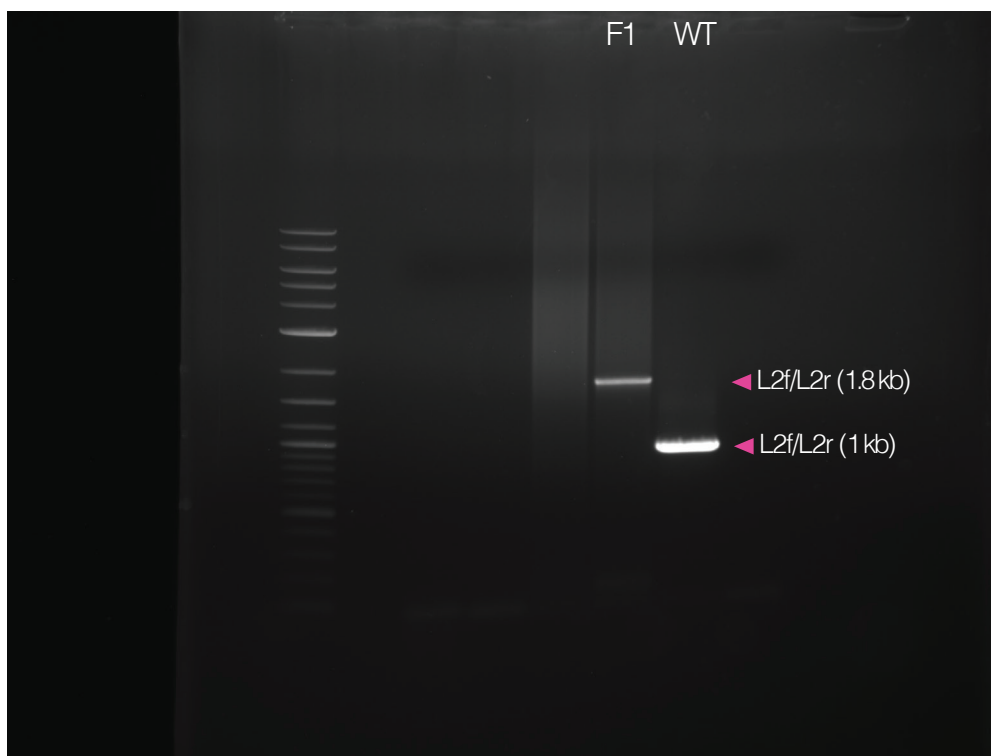

Supplement: Figure 2—figure supplement 1—source data 4. [file elife-80639-fig2-figsupp1-data4.pdf]

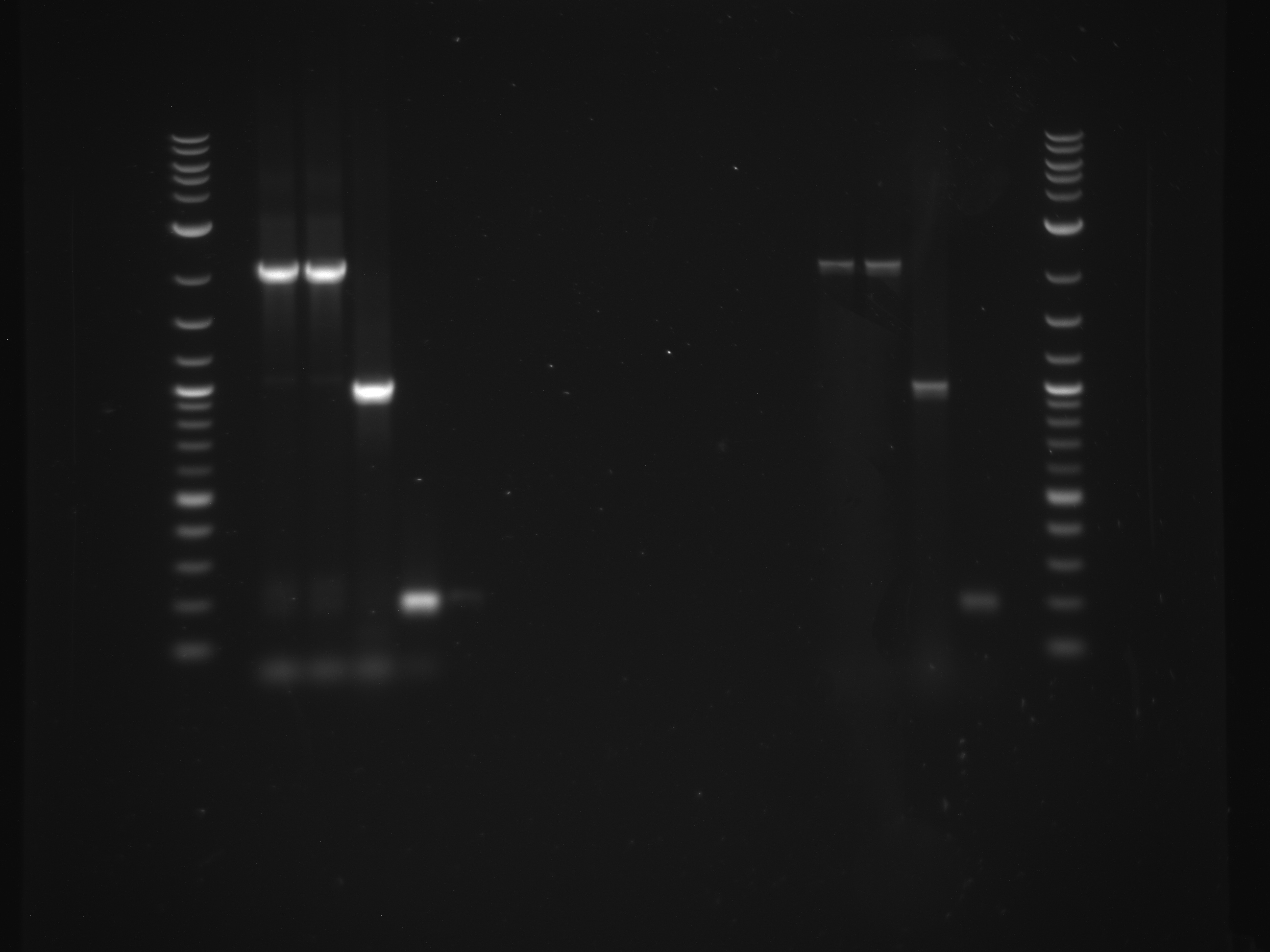

Supplement: Figure 3—source data 1. [file elife-80639-fig3-data1.zip › Figure3-Source Data1.jpg]

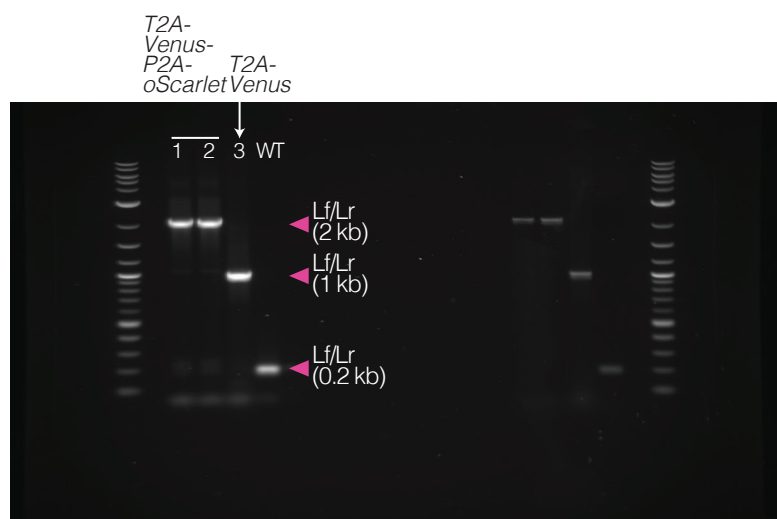

Supplement: Figure 3—source data 2. [file elife-80639-fig3-data2.pdf]

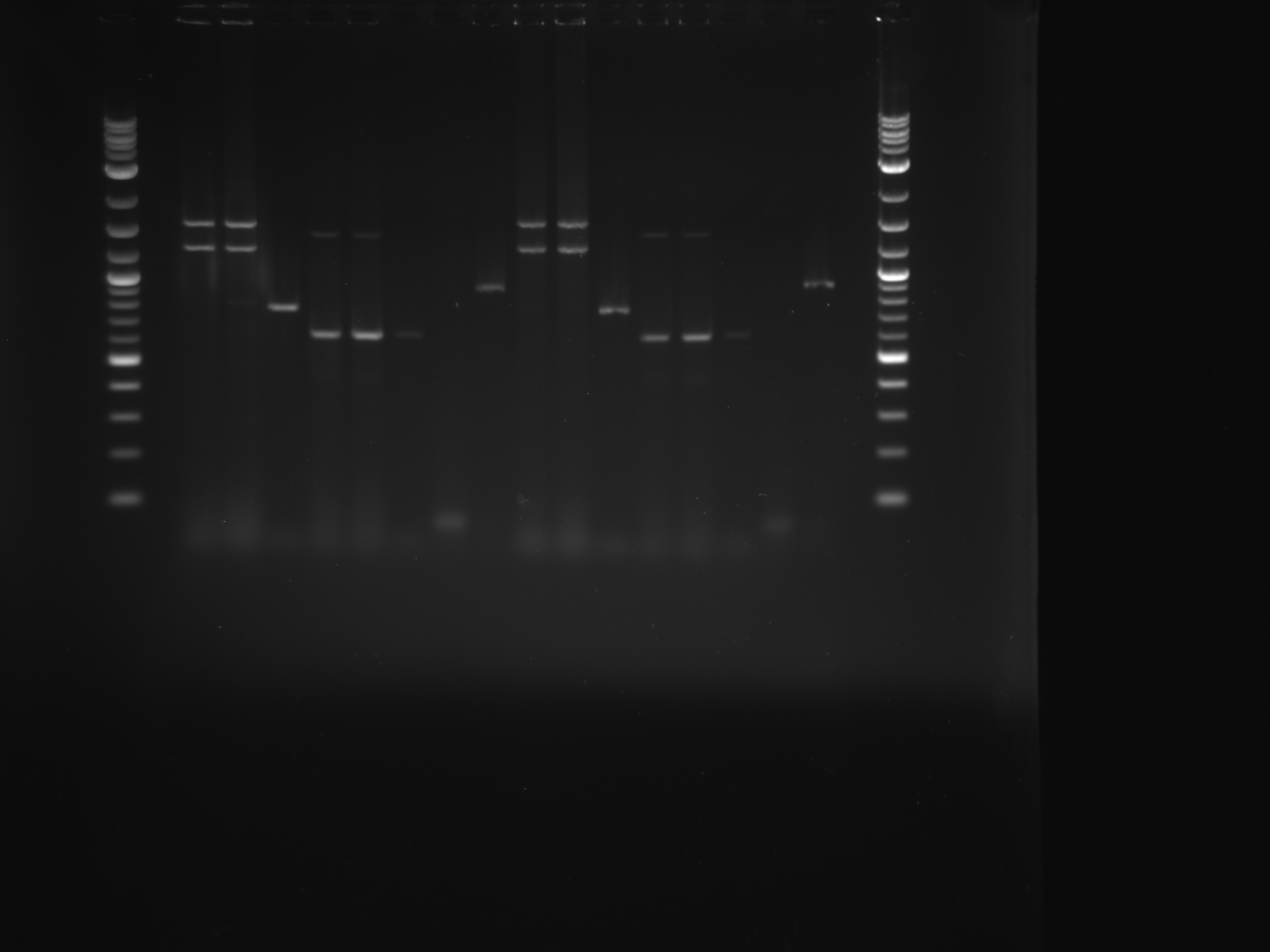

Supplement: Figure 4—source data 1. [file elife-80639-fig4-data1.zip › Figure4-Source Data1.jpg]

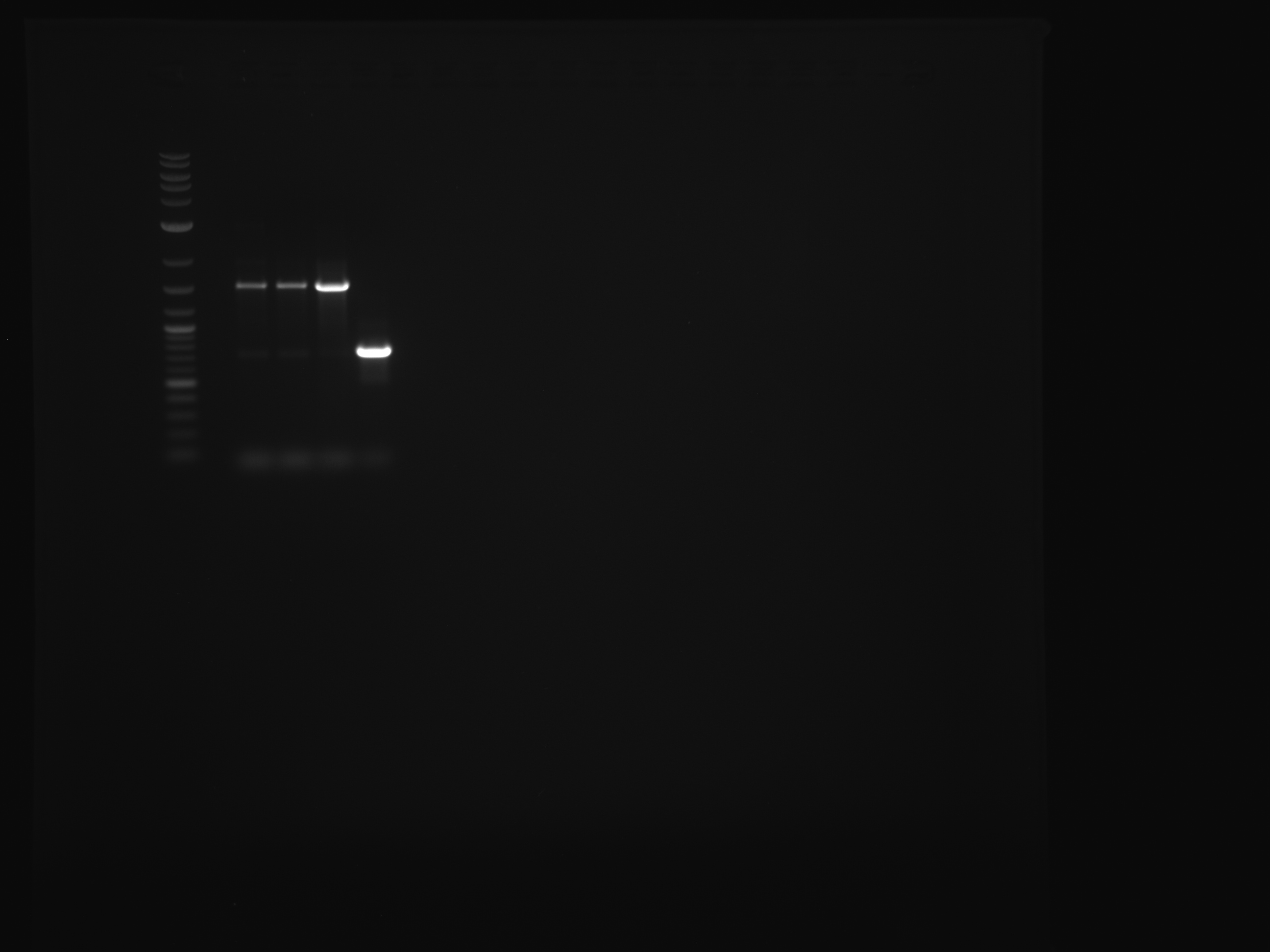

Supplement: Figure 4—source data 2. [file elife-80639-fig4-data2.zip › Figure4-Source Data2.jpg]

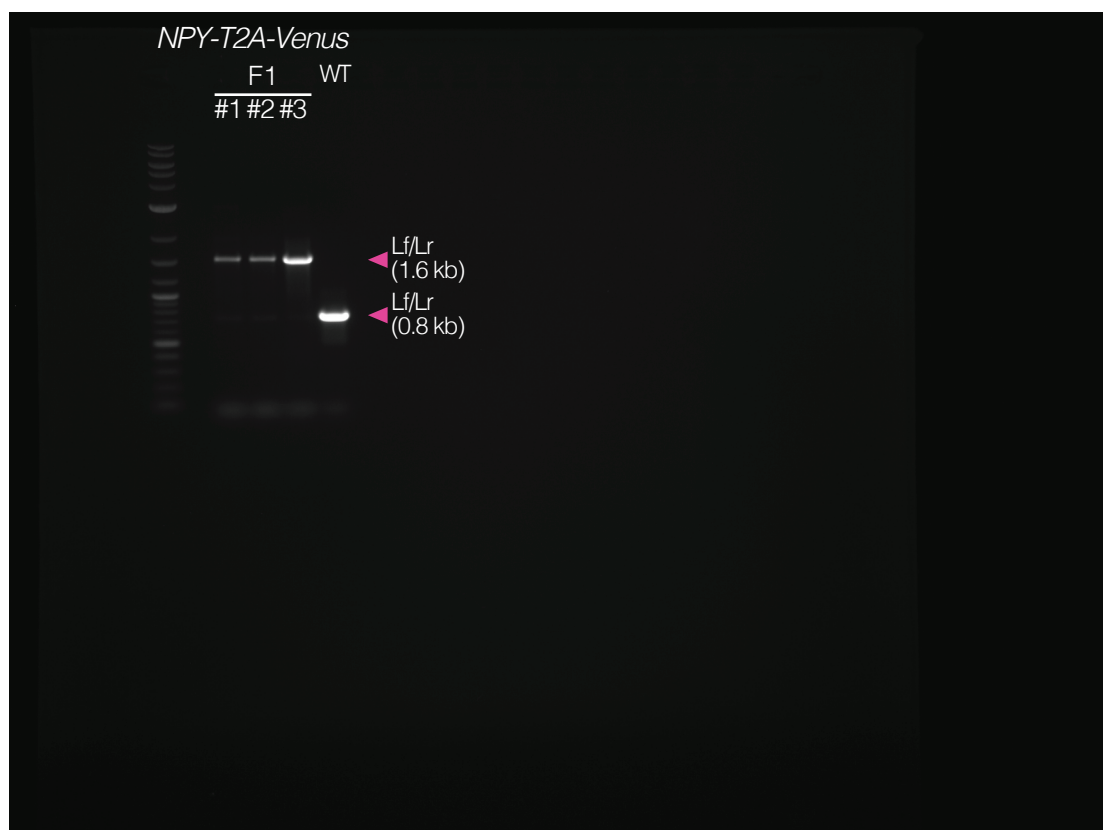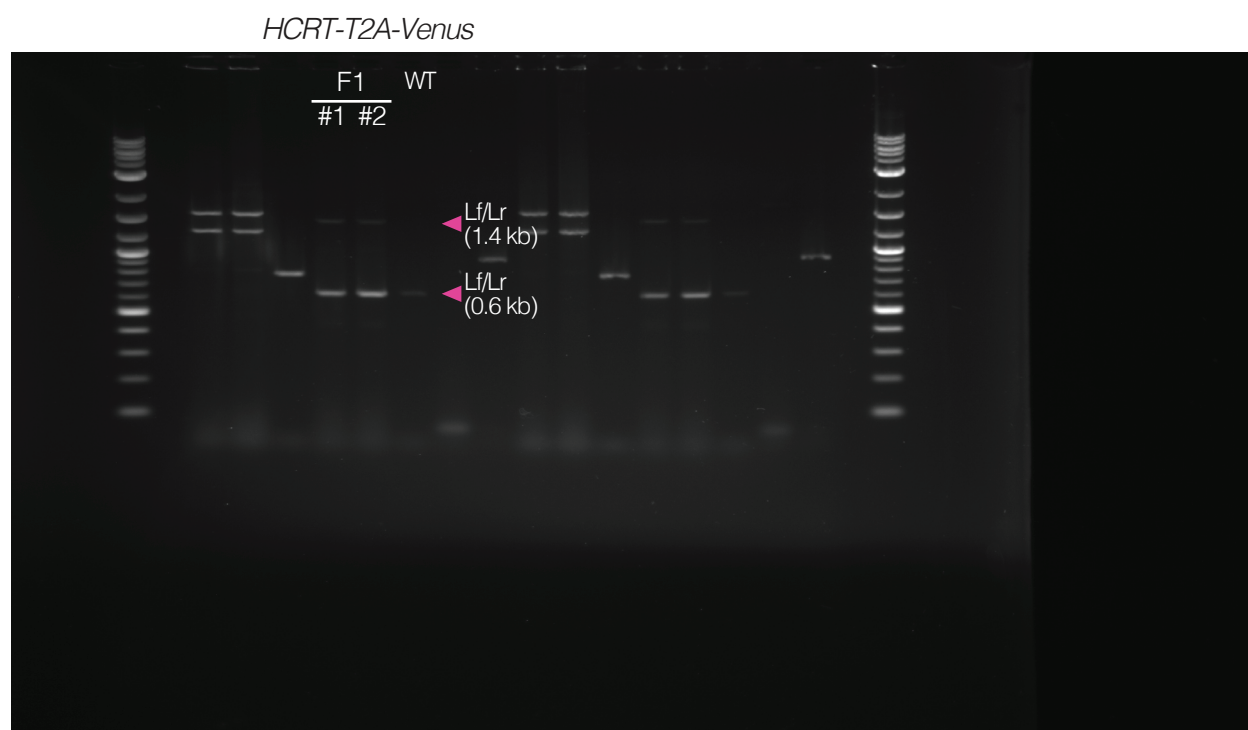

Supplement: Figure 4—source data 3. [file elife-80639-fig4-data3.zip › Figure4-Source Data3.pdf]

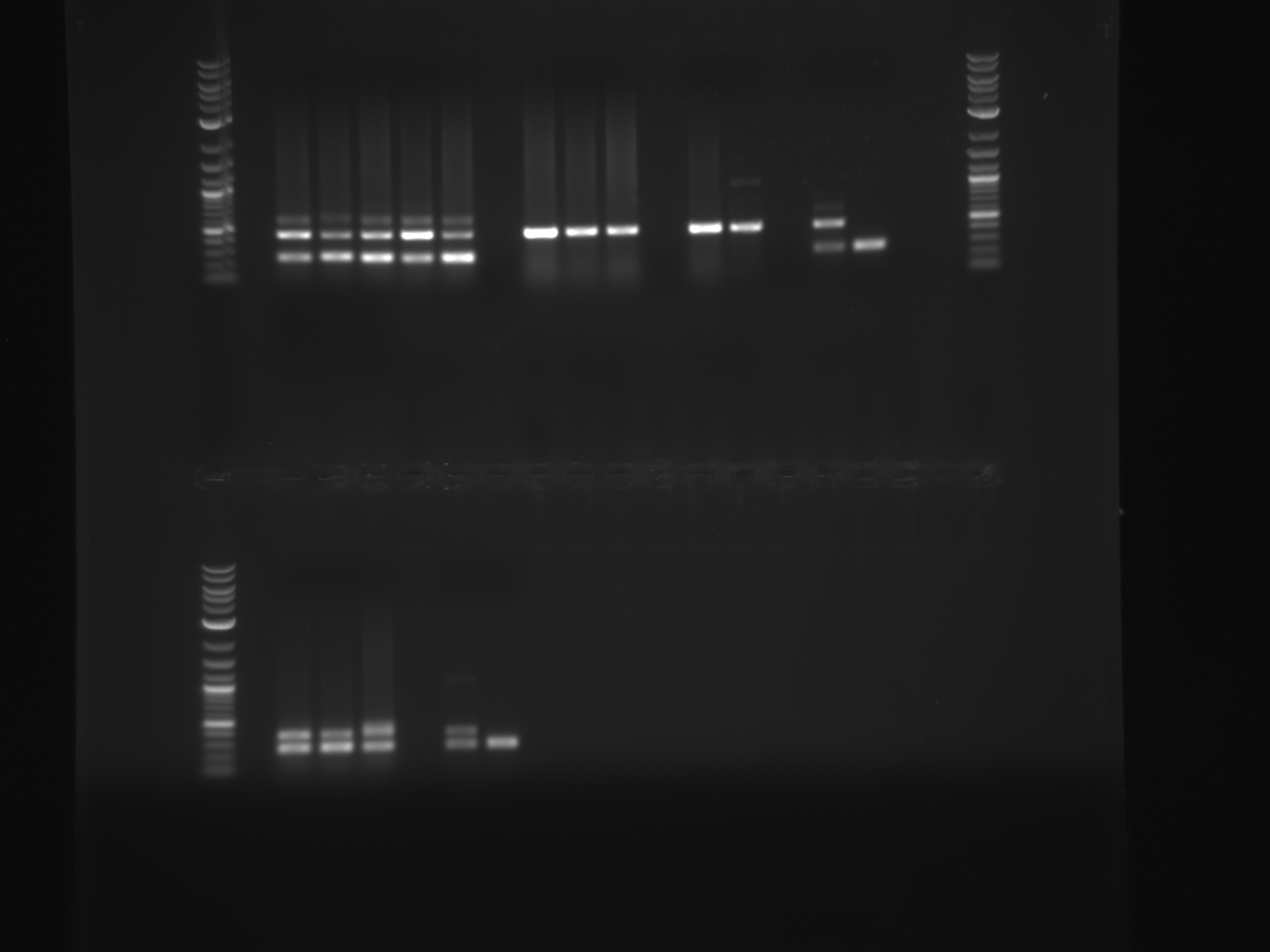

Supplement: Figure 4—figure supplement 1—source data 1. [file elife-80639-fig4-figsupp1-data1.zip › Figure4-figure supplement 1-Source Data1.jpg]
