# Supplementary material for: Rapid and precise genome engineering in a naturally short-lived vertebrate
Source: eLife. 2023 May 16;12:e80639. doi: 10.7554/eLife.80639 (PMC10188113; doi:10.7554/eLife.80639)
Supplement: Figure 4—figure supplement 1—source data 2. [file elife-80639-fig4-figsupp1-data2.zip › Figure4-figure supplement 1-Source Data2.pdf]

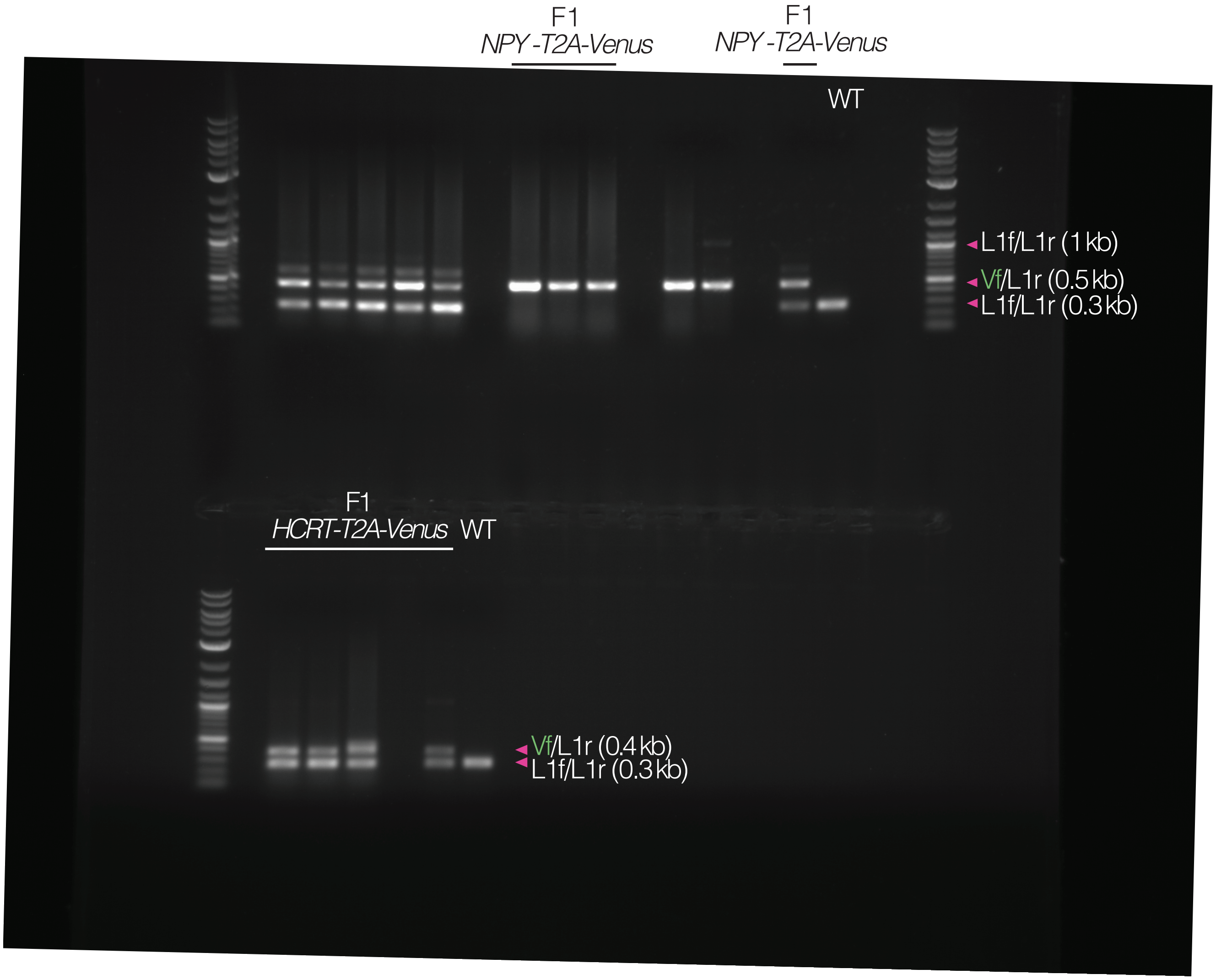
F1
NPY -T2A-Venus
F1
NPY -T2A-Venus
WT
L1f/L1r (1 kb)
Vf/L1r (0.5 kb)
L1f/L1r (0.3 kb)
F1
HCRT-T2A-Venus
WT
Vf/L1r (0.4 kb)
L1f/L1r (0.3 kb)
