# Supplementary material for: Rapid and precise genome engineering in a naturally short-lived vertebrate
Source: eLife. 2023 May 16;12:e80639. doi: 10.7554/eLife.80639 (PMC10188113; doi:10.7554/eLife.80639)
Supplement: Supplementary file 1. [file elife-80639-supp1.pdf]

CRISPR/Cas9-mediated knock-in for killifish

Introduction

This is a protocol for CRISPR/Cas9-mediated knock-in using all purchasable Integrated DNA Technologies (IDT) reagents utilizing the tracrRNA/crRNA technology, recombinant Cas9 (rCas9) protein, and IDT's HDR Enhancer V2. Using this protocol, we observe efficient whole-gene insertion in the killifish.

The following link contains information on ordering tracrRNA, crRNA, rCas9 and IDT's HDR Enhancer V2:
https://www.idtdna.com/pages/products/crispr-genome-editing/alt-r-crispr-cas9-system

Materials

- Alt-R® CRISPR-Cas9 tracrRNA (100 nmol; IDT, cat. no. 1072534)
  - IDT's standard RNA guide component for binding crRNA to rCas9 protein.
- Alt-R® CRISPR-Cas9 crRNA (2 nmol; IDT)
  - https://www.idtdna.com/pages/products/crispr-genome-editing/alt-r-crispr-cas9-system
  - Designed specifically for the desired genomic location to guide Cas9 to desired genomic location and make cut.
- Alt-R™ HDR Donor Block (3 µg; IDT)
  - https://www.idtdna.com/pages/products/crispr-genome-editing/alt-r-hdr-donor-blocks
  - Designed specifically for the knock-in of interest with homology arms for the genomic site of insertion.
  - HDR Donor Block product includes chemical modification.
  - Note: As an alternative, gBlocks™ Gene Fragments (IDT) can be used for the HDR template however these do not include chemical modification and have lower knock-in efficiency.
- Alt-R® S.p. Cas9 Nuclease V3 (rCas9; 500 µg; 10 µg/µl; IDT, cat. no. 1081059)
- Nuclease free (NF) duplex buffer (IDT, cat. no. 11-01-03-01)
  - Note: This buffer comes included with crRNA from IDT
- 1x PBS (Corning, cat. no. 21-040-CV)
- Alt-R™ HDR Enhancer V2 (150 µl of 0.69 mM in DMSO; IDT, cat. no. 10007921)
- Phenol red solution (0.5% wt/vol; Sigma-Aldrich, cat. no. P0290)

Procedure

Resuspend tracrRNA

1. Spin down lyophilized tracrRNA (i.e., Alt-R® CRISPR-Cas9 tracrRNA) using a benchtop centrifuge.
2. Resuspend tracrRNA in NF duplex buffer to 100 µM. Gently pipet up and down to resuspend. E.g., 100 nmol tracrRNA in 1 ml NF duplex buffer.
3. Divide the 100 µM dilution into ~20 µl aliquots to avoid excessive freeze-thaws.
4. Store tracrRNA at -20°C and work with on ice.

Resuspend crRNA

5. Spin down lyophilized crRNA.
6. Resuspend crRNA (i.e., Alt-R® CRISPR-Cas9 crRNA) in NF duplex buffer to 100 µM. Gently pipet up and down to resuspend. E.g., 2 nmol crRNA in 20 µl NF duplex buffer.
7. Store crRNA at -20°C and work with on ice.

Resuspend HDR donor template

8. Spin down lyophilized HDR donor template (i.e., Alt-R™ HDR Donor Block or gBlocks™ Gene Fragment).
9. Resuspend HDR donor template in NF duplex buffer to a concentration of 150 ng/µl. Gently pipet up and down to resuspend. E.g., 3 µg Alt-R™ HDR Donor Block in 20 µl NF duplex buffer.
10. Store at -20°C and work with on ice.

Dilute HDR Enhancer V2

11. Make working solution of HDR enhancer V2 (i.e., Alt-R™ HDR Enhancer V2) by diluting stock (0.69 mM) in NF duplex buffer to 10 µM. E.g., 1 µl of 0.69 mM HDR enhancer V2 stock in 68 µl of NF duplex buffer.

Prepare gRNA mix

12. Mix the following:

Reagent	Volume (µl)
tracrRNA (100 µM)	1
crRNA (100 µM)	1
NF duplex buffer	31.3

13. To anneal, heat mixture to 95°C for 5 min. This can be done using a PCR machine.

Prepare the ribonucleoprotein (RNP) complex

14. Mix the following:

Reagent	Volume (µl)
gRNA mix	10
rCas9 (10 µg/µl)	0.5
1x PBS	5.5

15. Heat mixture to 37°C for 10 min. This can be done using a PCR machine.

Prepare the injection mixture

16. Mix the following:

Reagent	Volume (µl)
RNP complex	8
HDR donor template (150 ng/µl)	1
HDR Enhancer V2 (10 µM)	1

17. To make the final injection mixture, add 0.33 µl of 0.5% Phenol red. Too much Phenol red can cause clogging of the injection needle. Only add as much is necessary to see the red color. Store injection mixture on ice until ready for injection and use within 1 hr of preparation.
